# An Account of Mr. Hunter's Method of Performing the Operation for the Popliteal Aneurism

**Published:** 1786

**Authors:** Everard Home

**Affiliations:** Surgeon


### IV. *An Account of Mr.* Hunter's *Method of performing the Operation for the Popliteal Aneurism. Communicated in a Letter to Dr.* Simmons *by Mr.* Everard Home, *Surgeon.*

*To Dr.* Simmons.

SIR,

IN consequence of your request, I send you an account of Mr. Hunter's method of performing the operation for the popliteal aneurism, which you will please to insert in the next part of the Medical Journal, if you think it deserving of a place in that publication.

I am, Sir,

Yours, &c.

Everard Home.

The common method of operating in cases of popliteal aneurism having, in many instances, proved unsuccessful, the operation itself has been condemned by some of our most eminent surgeons.

If we consider the cases in which it has been performed, and where the patients have died, we shall probably find that in all of them the artery had been diseased at the part enclosed by the the ligature, and had either ſloughed off, or had been cut through where it was tied, ſo that the ſides of the artery, though brought together, had not remained a ſufficient length of time in that ſituation to unite by the firſt intention, and the patients loſt their lives from the conſequent hæmorrhage.

The femoral and popliteal arteries are portions of the ſame trunk, preſenting themſelves on different ſides of the thigh, and are readily come at in either ſituation; but where the artery is paſſing from the one ſide to the other, it is more buried in the ſurrounding parts, and cannot be expoſed without ſome difficulty. In performing the operation for the popliteal aneuriſm, eſpecially when the tumour is large, the ligature is commonly applied on the artery at that part where it emerges from the muſcles. This will be too limited a ſpace, ſhould it prove diſeaſed for ſome way higher up; and if the artery ſhould afterwards give way from any of the cauſes above mentioned, there will not be a ſufficient length of veſſel remaining to allow of its being again ſecured in the ham. To follow the artery up through the inſertions of the Triceps muſcle, to get at a portion of it where it is found, becomes a very diſagreeable part part of the operation; and to make an incifion upon the fore part of the thigh, to get at and fecure the femoral artery, would be breaking new ground—a thing to be avoided, if poffible, in all operations.

From thefe confiderations, fuggefted by the accident of the artery giving way, which happened feveral times to Mr. Hunter, he propofed, in performing this operation, that the artery fhould be taken up at fome diftance from the difeafed part; fo as to diminifh the rifk of hæmorrhage, and admit of the artery being more readily fecured, fhould any fuch accident happen. The force of the circulation being thus taken off from the aneurifmal fac, the caufe of the difeafe would, in Mr. Hunter's opinion, be removed; and he thought it highly probable that if the parts were left to themfelves, the fac, with the coagulated blood contained in it, might be abforbed, and the whole of the tumour removed by the actions of the animal œconomy, which would confequently render any opening into the fac unneceffary.

The operation was firft performed in this way at St. George's hofpital, and the refult which I have here annexed does credit to Mr. Hunter's obfervation; and fo far as one cafe can eftablifh

 a general a general practice, it seems to be an improvement of considerable importance.

A. B. about forty-five years of age, a coachman, was admitted into St. George's hospital in December, 1785, with a popliteal aneurism, which he had perceived for three years, and had observed it gradually to increase during the whole of that period. It was so large as to distend the two ham strings laterally, and make a very considerable rising between them; the pulsation was very distinct, and could be felt on every side of the tumour. That leg and foot were so much swelled as to be a great deal thicker than the other, and were of a mottled brown colour; the swelling was not of the œdematous kind, but felt firm or *brawny*, being a consequence of the extravasation of coagulating lymph, so that the leg retained its natural shape.

Mr. Hunter having determined to perform the operation, a tourniquet was previously applied, but not tightened, that the parts might be left as much in their natural situation as possible; and he began the operation by making an incision on the fore and inner part of the thigh, rather below its middle, which incifion was continued obliquely acrofs the lower edge of the fartorius mufcle, and was made large to give room for the better performing of whatever might be neceffary in the courfe of the operation: the fafcia, which covers the artery, was then laid bare for about three inches in length, and the artery being plainly felt, a flight incifion, about an inch long, was made through this fafcia along the fide of the veffel, and the fafcia diffected off, by which means the artery was expofed. Having difengaged the artery from its lateral connexions by the knife, and from the parts behind it by means of the end of a thin fpatula, a double ligature was paffed behind it by means of an eyed probe, and the artery tied by both portions of the ligature, but fo flightly as only to comprefs its fides together; a fimilar application of ligature was made a little lower; and the reafon for paffing four ligatures was to comprefs fuch a length of artery as might make up for the want of tightnefs, as he chofe to avoid great preffure on the veffel at any one part. The ends of the ligatures were carried directly out at the wound; the fides of which were now brought together, and fupported by fticking plafter and a linen

 roller, roller, that they might unite by the firft intention.

The limb was found, fome hours after the operation, not only to retain its natural heat, but to be even warmer than the other leg. The fecond day after the operation the brawny firmnefs of the leg was confiderably diminifhed; it was become foft, loofe, and a good deal fmaller, and the aneurifmal tumour appeared to have loft more than one third of its fize.

Nothing could fhew more plainly the action of the abforbents than the change the leg underwent in fo fhort a time. The diminution of the tumour probably arofe from the fluid blood which it contained having paffed into collateral branches, or the tibial artery.

The fourth day, on the removal of the dreffings, the edges of the wound were found united through its whole length, excepting where prevented by the ligatures; there was neither pain nor tumefaction in the part, but the aneurifmal tumour was much the fame as on the fecond day.

On the ninth day after the operation there was a confiderable difcharge of blood from the part where the ligatures paffed out; a tourniquet was therefore applied on the artery above, which which ſtopped the bleeding; and although the tourniquet was taken off a few hours after, no blood followed. The head of a roller was now placed upon the wound, in the direction of the artery, and over that the tourniquet, which was not tightened more than was thought ſufficient to take off the impetus of the blood in that portion of the artery.

On the tenth day appearances were much the ſame, only that between the compreſs and the knee there appeared a little fulneſs like an approaching inflammation. On the eleventh day this was gone off, and on the fifteenth ſome of the ligatures came away, followed by a ſmall diſcharge of matter, and the tumour in the ham was leſſened. On the ſeventeenth day the parts ſurrounding the aneuriſmal tumour were more reduced and pliable, ſo that it became diſtinct.

About the latter end of January, 1786, ſix weeks after the operation, the patient went out of the hoſpital, the tumour at that time being ſomewhat leſſened, and rather firmer to the feel. He was ordered to come to the hoſpital once every week, and, in the mean time, to make ſome degree of preſſure by applying a compreſs and bandage, with a view to excite the the abſorbents to action, which, in this as in moſt caſes, had a good effect.

About the middle of February the tumour had decreaſed, and was become ſtill firmer. March the 8th the wound, which had cicatrized, broke out again, and the patient was taken into the hoſpital. About the 8th of April ſome remaining threads of the ligature came away, and an inflammation appeared upon the upper part of the thigh. In the middle of May a ſmall abſceſs broke at ſome diſtance from the old cicatrix, at which opening ſome matter was diſcharged, but no pieces of ligature were obſerved. Several ſmall threads were, at different times, diſcharged at the old ſore, and the ſwelling ſubſided; but the thigh ſoon ſwelled again to a greater ſize than before, attended with conſiderable pain. In the beginning of July a piece of ligature, about an inch in length, came away; after which the ſwelling ſubſided entirely, and he left the hoſpital the 8th day of July, at which time there remained no tumour in the ham, and he was in every reſpect well.

This mode of performing the operation being in itself evidently more fimple, and in every refpect lefs dangerous than the method commonly practifed, it is unneceffary to enumerate all the circumftances in which it deferves the preference: but, before I conclude, it will be proper to obferve, that Mr. Hunter now rather difapproves the application of a number of ligatures, in the manner practifed in the above cafe, as thefe cannot come away without producing ulceration on that part of the artery which they enclofe, a tedious procefs when the ligature is not drawn tight; neither do I believe he would again be inclined to heal up the wound by the firft intention, but rather to allow the cut furface to inflame and fuppurate, by which he would have it more in his power to come at the artery, fhould that prove neceffary; and probably, by means of the dreffings, he might make a gentle compreffion to affift the ligatures.

It will not be improper here to obferve, that furgeons have laid too much ftrefs on the neceffity of large, collateral branches being prefent, to infure the fuccefs of this operation: this muft have arifen more from their anatomical knowledge, than from obfervations made from practice, practice, since we find that the trunk of the femoral artery may be taken up in any part of the thigh, without producing mortification of the limb. In one patient afflicted with aneurism, whose limb Mr. Hunter examined after his death, though there was great reason to believe that the artery had been obliterated above the great muscular branch, the limb had been very well nourished.

Since the above account was drawn up, this mode of performing the operation has been practised, in a case of aneurism of the femoral artery, by Mr. Birch, surgeon to St. Thomas's hospital; and as the operation, in that instance, was not attended with success, the failure might by some be attributed to the mode in which it was performed, and the above account might be considered as a partial one; to do away these objections, I requested Mr. Birch to favour me with the particulars of the case, that my account of the consequences of the operation might be more complete; and although it was disagreeable to me to ask for the recital of an unsuccessful case, I was well assured that Mr. Birch was too disinterested, and too anxious to promote the improvement of his profession, to make the least objection to the case case being laid before the public: and I have since been fully confirmed in my opinion by his ready compliance with my request.

I have given the case, with the dissection of the body after death by Mr. Cline, exactly as they were communicated to me by Mr. Birch.

"CASE.

"John Lewis, a negro, aged forty-three "years, received a blow on the anterior part "of the right thigh. About a month after "he perceived a small tumour, which gradu-"ally increased, and his own expression was, "that he could feel it *thump, thump*.

"As the tumour enlarged, he came to Lon-"don for advice, applied at St. Thomas's "hospital on Thursday the 26th of Octo-"ber last, and was directly admitted. On "examination, I found a large tumour, ex-"tending within two inches of Poupart's li-"gament upwards, and occupying two thirds "of the thigh; a pulsation could be felt, and "there was no doubt of the disease being an "aneurism of the femoral artery.

"I directed seven ounces of blood to be "taken from the arm, and an opiate to be

" given at night. The patient refted well,

" and the next day a confultation was held, in

" which it was propofed to perform an opera-

" tion, and endeavour to pafs a ligature round

" the femoral artery, giving the patient the

" chance of nourifhing the limb by the arteria

" profunda, and other anaftamofing veffels.

" On Friday the 3d of November it was de-
" termined to perform the operation. Mr.
" Cline undertook to comprefs the artery as it
" paffed through Poupart's ligament, which
" he eafily effected with a hard comprefs in the
" fhape of a T with a broad bafis.

" It was agreed, previous to the operation,
" that an incifion fhould be carried in a femi-
" lunar form round the upper part of the
" aneurifmal fac, in order to make room for
" the longitudinal incifion neceffary to diffect
" down to the artery. This was accordingly
" done, and the integuments raifed, fo as to
" make room to feel for the pulfation of the
" artery. Some portion of cellular membrane,
" and fome lymphatic glands, were neceffarily
" diffected and removed; with my fingers I
" then feparated the mufcular fibres, and tore
" away the connecting parts, till the artery
" could be plainly felt in pulfation. It was

" then

" then neceſſary to divide a part of the faſcia

" covering the artery, which was done by car-

" rying the back of the knife on Mr. Cline's

" nail, while his finger preſſed upon the naked

" artery; after which the finger and thumb

" could ſurround and compreſs the veſſel.

" An eye probe, armed with a ſtrong flat liga-

" ture, was then puſhed through the cellular

" membrane, and carried under the artery.

" This being effected, we had ſuch command

" of the veſſel as to be able to ſtrip it down,

" and paſs another ligature ſomewhat lower.

" This laſt ligature was then tied, the firſt

" being left looſe to ſecure us againſt accident.

" The threads being ſeparated and ſecured,

" the wound was lightly dreſſed, the tumour

" left in its natural ſituation, and the patient

" put to bed, with the loſs of only four or five

" ounces of blood during the operation. No

" pulſation could be perceived in the tumour

" after the ligature was tied.

" On Saturday, November 4th, he had ſlept

" well, was eaſy, and there was ſufficient

" warmth in the extremity to aſſure me of ſome

" circulation.

" On the 5th the diſcharge from the wound-

" ed lymphatics was ſo abundant as to make

" it neceſſary to remove the ſuperficial dreſ-

" ſings. The tumour was rather ſofter to the

" touch, and the ſkin about the apex of it be-

" gan to ſhrivel.

" The diſcharge of lymph continued till the

" 9th, and then the wound began to digeſt,

" affording, however, a very ſmall quantity

" of pus. The tumour grew thinner at one

" point, and ſeemed as if diſpoſed to ulcerate

" the integuments. This day I paſſed a bleed-

" ing ligature round the leg, juſt below the

" knee, and the veins tumefied ſufficiently to

" have bled freely, if they had been punc-

" tured.

" 10th, He was feveriſh in the evening.

" 11th, He had ſtools from ſome laxatives

" I had directed, and was better.

" 12th, The tumour was very thin in one

" part, and a fluctuation evidently to be felt.

" The limb was warm and moveable, but the

" patient was feveriſh and delirious at night.

" A decoction of bark, with a ſedative bolus,

" were directed for him, but he would not

" take them.

" 13th, The wound looked florid, and af-

" forded good pus. The patient was feveriſh

" and

" and delirious; the tumour was threatening

" to burſt. This day he took his medicines.

" 14th, He became ſenſible, but was lan-

" guid and hot; the tumour burſt, and diſ-

" charged ſerum and grumous blood; he

" fainted; the dreſſings were not diſturbed;

" he ſlept compoſedly; fainted again about

" ſix o'clock in the evening, and expired. I

" ſaw him at ſeven, when the limb was ſtill

" warm; I removed the dreſſings, and found

" a ſmall ſtream of freſh arterial blood, which

" had iſſued from the wound.

" It appears probable, that if the patient

" had applied for relief before the tumour was

" ſo much enlarged, the operation might have

" ſucceeded, as we ſhould then have been able

" to have tied the ſound artery ſo much lower

" down.

" J. BIRCH.

" DISSECTION.

" The body was examined the morning after
" the patient's death. The integuments on
" the middle of the tumour were mortified.
" The blood contained in the tumour was
" very putrid, and the greater part of it fluid;
" it appeared to be diſſolved by putrefaction.

" Water,

"Water, injected by the external iliac ar-
"tery, escaped freely from the wound at the
"ligature, where the artery was open and
"appeared to have ulcerated at that part.

"In dilating the artery from the ligature to

"the heart, its internal surface appeared of a

"bright red. This appearance lessened at the

"curvature of the aorta, yet it was very evi-

"dent in its semilunar valves.

"The arteria profunda, which passed off

"from the femoral artery rather less than half

"an inch above the ligature, was also inflamed

"within. There were near two inches of the

"femoral artery between the ligature and the

"aneurismal sac, the internal surface of which

"was of the usual white colour; from this a

"membranous-like substance could be peeled

"off, that seemed to be coagulable lymph.

"The opening, where the artery passed out

"of the aneurismal sac, was near three inches

"below the part where it entered. In opening

"this part of the artery, from the sac to the

"ham, it appeared quite sound, and of its

"natural colour.

"H. CLINE."

